# Community-level determinants of loneliness and social isolation: a population-based cohort study across younger and older adults

**DOI:** 10.3389/fpubh.2025.1526166

**Published:** 2025-05-15

**Authors:** Drew Eleanor Meehan, Philip Clare, Anne Grunseit, Dafna Merom

**Affiliations:** ^1^School of Health Sciences, Western Sydney University, Campbelltown, NSW, Australia; ^2^Prevention Research Collaboration, University of Sydney, Sydney, NSW, Australia; ^3^National Drug and Alcohol Research Centre, UNSW Sydney, Sydney, NSW, Australia; ^4^Charles Perkins Centre, University of Sydney, Camperdown, NSW, Australia; ^5^School of Public Health, Faculty of Health, University of Technology Sydney, Sydney, NSW, Australia

**Keywords:** loneliness, social isolation, public health, community-level, longitudinal

## Abstract

**Introduction:**

Loneliness and social isolation (SI) are critical public health issues with well-documented effects on health and well-being. However, much of existing observational and intervention research has focused predominantly on individual-and interpersonal-level factors. This longitudinal study addresses significant knowledge gaps by comprehensively examining the independent influence of multiple community-level determinants on loneliness and SI and uniquely comparing these effects across younger (18–30 year) and older (60 + years) adults within an Australian population cohort over a 12-year period.

**Methods:**

Using longitudinal data from the Household, Income and Labour Dynamics in Australia (HILDA) survey, we analysed data from four wave pairs (2006/07, 2010/11, 2014/15, 2018/19) to investigate associations between loneliness and SI and nine community and neighbourhood-level variables. We employed lagged mixed-effects Poisson regression models to calculate risk ratios (RR) adjusted for individual-and interpersonal-level factors.

**Results:**

Our findings reveal that low community engagement is the strongest risk factor for loneliness and SI in both younger (Loneliness, RR = 1.34; SI, RR = 1.58) and older populations (Loneliness, RR = 1.35; SI = 2.02). Low neighbourhood social cohesion was found to significantly increase loneliness and SI in older adults (Loneliness, RR = 1.15; SI, RR = 1.36) and to increase SI in younger adults (RR = 1.54). We also observed distinct age-specific effects, with cultural practices, altruism, and perceived neighbourhood safety having differential impacts across age groups.

**Discussion:**

Our findings highlight the critical need for community-level interventions to address loneliness and SI, suggesting that focusing solely on individual-related factors is insufficient. Tailoring public health strategies to enhance community dynamics may be essential in reducing loneliness and SI among vulnerable populations, particularly in areas with low social cohesion and community engagement offerings.

## 1 Introduction

The effect of loneliness and social isolation (SI) on the population’s health are well established. Clear links have been found between loneliness and an increased risk of cardiovascular disease, dementia, and mental ill-health (1–3). Similarly, SI can lead to poorer mental health, lower self-perception of health and the adoption of unhealthy behaviours such as a sedentary lifestyle (2, 4). Additionally, there is evidence for considerable economic costs associated with loneliness and SI, although this research is still in its infancy (5).

Given these significant health impacts, there is a need for clear definitions and frameworks to guide the research that we conduct, also helping to reduce the currently siloed nature of the research into loneliness and SI (6). Loneliness is defined for the purposes of this study as ‘the subjective unpleasant or distressing feeling of a lack of connection to other people, along with a desire for more, or more satisfying, social relationships’ while SI refers to having ‘objectively few social relationships, social roles, group memberships, and infrequent social interaction’ (7). The two may be viewed as a matrix, whereby people may be lonely but not socially isolated, or conversely isolated but not lonely, both or neither. It has been argued that the two experiences, while distinct, are closely entwined and therefore should be investigated in tandem (8).

Typically, loneliness and SI have been investigated and intervened upon primarily at the individual and interpersonal level, overlooking the benefits of a population approach and resulting in interventions with mixed efficacy (9). Therefore, in line with previous research, we propose that an appropriate framework for the investigation of loneliness and SI is the social ecological model (6). This model represents a diversion from the typical individualistic approach to health, to a more holistic view of health determinants (10). While there are numerous versions of the social ecological model, the one that we have used to conceptualise this study consists of four nested circles representing the four levels of influence on peoples’ experiences of loneliness and SI, namely the individual, interpersonal, community and societal levels (11). This model is particularly relevant for a public health approach to loneliness and SI, as it contextualises how community and societal factors influence social connection beyond individual choices (12).

Community-level interventions are commonplace in public health and have the potential to influence behaviour at the population level with greater exposure (13). The majority of studies which have investigated the broader community-level determinants of loneliness and SI have only focused on one or two factors at a time, and commonly used cross-sectional designs, hence a more comprehensive approach is needed (6, 14). This would support more comprehensive polices and help to guide the development of more effective interventions.

While younger and older adults are the two groups found to be most at risk of experiencing loneliness and SI (4, 15), there is little research exploring the similarities and differences of the various community-level determinants within these cohorts (6, 14). This limits the ability to identify the common factors for intervening at the community-level, and those more idiosyncratic for a particular age group. Understanding the differential effects of community-level factors on the experience of loneliness and SI in younger and older people would help to create targeted interventions addressing the unique contexts of each population. Therefore, we aim to use a longitudinal study design to identify community-level determinants of loneliness and SI and compare their effects among younger and older adults within a population cohort in Australia.

## 2 Methods

### 2.1 Data source

We used data from the Household, Income and Labour Dynamics in Australia (HILDA) survey, a nationally representative longitudinal panel survey. A detailed description of the HILDA survey design has been published previously by Wooden and Watson (16). In short, the HILDA sample is a multi-stage stratified random sample of Australian private dwellings (*n* = 7,682 households in 2001, the Wave 1-baseline sample), in which household members over the age of 15 were recruited into a panel (17). The household response rate to the baseline survey was 66%. Panel members have been subsequently reinterviewed annually and in 2011, a top-up sample of 2,153 households was added to ensure the survey remained nationally representative (17). A range of household, economic and labour questions are asked in interviews with participants. A self-completion questionnaire with more sensitive questions is then offered to participants and could be completed online or on paper and mailed back to the researchers (18).

Due to the rotating nature of the questions used in the HILDA surveys, the data required to address our research question were collected only every 4 years. Consequently, we use data from Waves 6–19 (2006–2019), where Wave 6, 10, 14 & 18 are used for covariates, and Waves 7, 11, 15 & 19 are used for loneliness and SI data. We have reported according to the Strengthening the Reporting of Observational Studies in Epidemiology (STROBE) statement (19), with the checklist available in Supplementary File 4.

### 2.2 Study sample

The study sample included only participants who are aged 18–30 (younger adults) or 60 + (older adults). Participants were included if they responded to the survey in Waves 6, 10, 14 and/or 18 and they answered all the questions included in the loneliness and/or the SI measure in the subsequent lagged wave. Consequently, if someone responded to Wave 6, they would also have needed to respond to the loneliness or SI questions in Wave 7 to be included.

### 2.3 Study measures

#### 2.3.1 Outcome variables

The loneliness and SI measures and scoring methods use those from a previous cross-sectional analysis of the HILDA data (20), and as detailed below. Additionally, these measures have been psychometrically tested using this data and these populations (21).

Loneliness: Three questions assessed loneliness by asking participants to their level of agreement with the statements ‘People do not come to visit me as often as I would like’, ‘I often need help from other people but cannot get it’ and ‘I often feel very lonely’ on a seven-point Likert scale (increasing scores denote increasing agreement). We dichotomised this variable into a lonely and not lonely group, using a median score of ≥4 across the three questions to denote loneliness as done previously (20).

Social isolation (SI): The statements used to assess SI were ‘there is someone who can always cheer me up when I’m down’, ‘I enjoy the time I spend with the people who are important to me’, ‘when somethings on my mind, just talking with the people I know can make me feel better’ and ‘when I need someone to help me out, I can usually find someone’. The questions were scored on a seven-point Likert scale, where a higher score denotes increasing agreement. Consistent with the construction of the loneliness variable, the SI variable was dichotomised, using a median score of ≤4 across the four questions to indicate the participant was experiencing SI (20).

#### 2.3.2 Independent variables

Community participation variables included in analyses comprise civic engagement, community engagement, altruism, and cultural practices. Neighbourhood variables were neighbourhood safety, neighbourhood social cohesion, neighbourhood atmosphere, remoteness, and Socioeconomic Index for Area (SEIFA) quintiles. All independent variables were treated as time-variant in our analysis which is further detailed in the Statistical Analysis section below. Descriptions of each variable are provided in Table 1. These community participation and neighbourhood variables were selected based on a previous review identifying the potential role of community factors in social connection outcomes (6), and represent different dimensions of community life that may affect opportunities for meaningful social interaction. The combination of these variables allows for a comprehensive assessment of the community environment while minimising collinearity between related concepts. Further details about the question wording and measure construction are detailed in the Supplementary File 1.

**Table 1:** Community participation and neighbourhood variables.

Factor	Description
Community participation variables	
Civic engagement	The extent to which participants engage in activities that aim to influence the community and society at large.
Community engagement	How often participants engage in social activities.
Altruism	The extent to which participants actively contribute to the community and/or non-profit organisations.
Cultural practices	The participants participation in events and spiritual practices that are characteristic of their community.
Neighbourhood variables	
Neighbourhood safety	The participants perception of safety in their local neighbourhood.
Neighbourhood social cohesion	The participants perception of cohesion in their local neighbourhoods including shared values in the local area, as devised by Sampson et al. (43)
Neighbourhood atmosphere	The participants perception of the neighbourhood noise levels and the aesthetics.
Remoteness	Determined using the 2011 Australian Statistical Geography Standard Remoteness Areas (44) based on the postcode of a participant’s primary address, into three categories; major cities, inner regional and outer regional-very remote.
SEIFA quintiles	Determined from the Australian Bureau of Statistics 2011 Socioeconomic Index for Area (SEIFA) index of relative socio-economic advantage/disadvantage using the participant’s primary address (45). This variable was categorised as quintiles where higher scores indicate lower disadvantage.

#### 2.3.3 Covariates

Wave of survey was used as a continuous time variable to account for change in loneliness and SI over the four time points, whereby each wave represents a four-year interval between 2006 and 2018.

Individual-and interpersonal-level variables included in analyses were age, gender, ethnicity, marital status, level of educational obtainment, self-assessed health, number of people in dwelling, working status, and gross annual household income. The operationalisation of each variable, can be found in Supplementary File 1.

### 2.4 Statistical analysis

We report unweighted descriptive statistics for each study population across each wave of interest, using raw numbers and percentages.

We conducted a series of mixed effects modified Poisson regression models to investigate the relationship between loneliness, SI, and the independent variables of interest over time, stratified by the two age groups: younger (18–30) and older adults (60+). Our models included both fixed effects (all independent variables and covariates) and random effects (person ID) regressed on the outcome variables, respectively. Results are reported as risk ratios and their 95% confidence intervals. We used these modified Poisson regressions as an alternative to log binomial regression (22). This has been found to be an appropriate method to account for the intra-person correlations that occur when measurements are repeated on the same subject over time, while maintaining binary outcomes (22, 23). The use of mixed effects modelling also allows for an unbalanced panel (24), meaning that participants do not need to have answered the included questions at all four time points to have their data included in the analysis which accounts for case-level missingness. Independent variables and covariates were included as fixed effects in the models, with the person ID as a random intercept.

We carried out all analyses using ‘lagged’ models, whereby loneliness and SI were regressed on all independent variables and covariates from the previous wave. For example, loneliness and SI from Wave 7 were regressed on variables from Wave 6, while loneliness and SI from Wave 11 were regressed on the variables from Wave 10. By using a lagged model, we control for temporary experiences of loneliness and SI (which are less likely to affect health) while still allowing the direction of the relationship between variables to be detected. Additionally, we treated loneliness and SI as independent variables within the other’s model. That is, when loneliness is the outcome variable, SI from the preceding wave was used as an independent variable and vice versa which allows for us to estimate the influence of each of these experiences on the other within the lagged analysis. We carried out all statistical analysis in Stata v.18.5 (25).

Categorical data analysis was chosen to improve the interpretation of the results, where we could predict the outcome (lonely vs. not lonely, SI vs. not SI), using more meaningful increases and decreases in covariates than those that can be achieved using continuous variables thereby improving the interpretability of the results. We conducted sensitivity analyses on the suitability of the operationalisation of the variables to verify this method. We compared models with continuous/binary outcomes variables and with continuous/categorical covariates using the Bayesian Information Criterion (BIC) and determined that using categorical outcome variables was a better fit than using continuous outcome variables. Using categorical covariates, despite having a larger BIC, was acceptable when balanced with the benefits of interpretation. Further information can be found in Supplementary File 3. Additional analyses were conducted to determine the effect of missing values, with investigations of which variables were most affected and patterns of missingness. Missing data were handled through pairwise deletion, where participants were only included if they had valid data for loneliness and SI in each analysis, and data for at least one of the independent variables. For household income, we used the imputed values provided by HILDA data custodians, however no additional imputation was performed for other variables. As shown in our supplementary analyses (Supplementary File 2), missing data were minimal for most variables (<5%), with only neighbourhood safety having substantial missingness (11.4%) primarily due to ‘do not know’ responses. Sensitivity analyses indicated that patterns of missingness did not substantially influence our main findings. Further information about the missing values can be found in Supplementary File 2.

## 3 Results

### 3.1 Sample characteristics

There were 16,163 participants within scope for this study between the ages of 18–30 (*n* = 9,414) or aged 60 + (*n* = 6,749) who responded to the survey in Waves 6, 10, 14 and/or 18 with an average of two wave pairs per participant. The final analytical sample included 13,799 participants, comprising 7,802 younger people (18–30) and 5,997 older people (60+). There was a sample population of 2,984 younger people in Wave 6, with this number growing to 4,406 by Wave 18. In Wave 6 there were 2,873 older people included in the sample, while the sample population of older adults in Wave 18 was 4,816.

Further sample characteristics are presented in Tables 2, 3 for each wave.

**Table 2:** Unweighted descriptive statistics for the younger population (18–30) stratified by wave.

Factors	Wave			
	Wave 6	Wave 10	Wave 14	Wave 18
	*n* (%) (*N* = 2,984)	*n* (%) (*N* = 3,494)	*n* (%) (*N* = 4,623)	*n* (%) (*N* = 4,406)
Age				
18–21	1,028 (34.45%)	1,211 (34.66%)	1,352 (29.25%)	1,218 (27.64%)
22–24	719 (24.10%)	823 (23.55%)	1,138 (24.62%)	961 (21.81%)
25–27	649 (21.75%)	764 (21.87%)	1,080 (23.36%)	1,140 (25.87%)
28–30	588 (19.71%)	696 (19.92%)	1,053 (22.78%)	1,087 (24.67%)
Gender				
Male	1,456 (48.79%)	1,769 (50.63%)	2,301 (49.77%)	2,174 (49.34%)
Female	1,528 (51.21%)	1,725 (49.37%)	2,322 (50.23%)	2,232 (50.66%)
Ethnicity				
Australian, non-indigenous	2,339 (83.48%)	2,823 (85.96%)	3,623 (83.02%)	3,464 (83.89%)
Australian, indigenous	106 (3.78%)	143 (4.35%)	226 (5.18%)	252 (6.10%)
Main English-speaking country born	113 (4.03%)	112 (3.41%)	173 (3.96%)	155 (3.75%)
Others	244 (8.71%)	206 (6.27%)	342 (7.84%)	258 (6.25%)
Marital status				
Legally married or de facto	1,212 (43.24%)	1,538 (46.83%)	1,995 (45.71%)	1,912 (46.31%)
Separated or divorced	35 (1.25%)	32 (0.97%)	52 (1.19%)	41 (0.99%)
Widowed	1 (0.04%)	1 (0.03%)	1 (0.02%)	1 (0.02%)
Never married and not de facto	1,555 (55.48%)	1,713 (52.16%)	2,316 (53.07%)	2,175 (52.68%)
Level of educational obtainment				
Tertiary level educated	569 (20.30%)	582 (17.72%)	922 (21.13%)	953 (23.08%)
Trade certificate	700 (24.97%)	815 (24.82%)	1,251 (28.67%)	1,185 (28.70%)
High school certificate	927 (33.07%)	1,196 (36.42%)	1,421 (32.56%)	1,354 (32.79%)
Did not finish high school	569 (20.30%)	664 (20.22%)	712 (16.32%)	581 (14.07%)
Still in school	38 (1.36%)	27 (0.82%)	58 (1.33%)	56 (1.36%)
Self-assessed health				
Excellent	404 (17.38%)	465 (17.16%)	616 (17.01%)	563 (16.01%)
Very good	975 (41.95%)	1,137 (41.96%)	1,521 (42.00%)	1,412 (40.15%)
Good	721 (31.02%)	882 (32.55%)	1,145 (31.62%)	1,184 (33.67%)
Fair	193 (8.30%)	206 (7.60%)	299 (8.26%)	299 (8.50%)
Poor	31 (1.33%)	20 (0.74%)	40 (1.10%)	59 (1.68%)
Number of people in dwelling				
Multi-person household	2,524 (84.58%)	3,051 (87.32%)	3,946 (85.36%)	3,794 (86.11%)
Lone person household	460 (15.42%)	443 (12.68%)	677 (14.64%)	612 (13.89%)
Working status				
Employed	2,250 (80.27%)	2,538 (77.26%)	3,263 (74.75%)	3,234 (78.32%)
Unemployed, looking for work	159 (5.67%)	224 (6.82%)	345 (7.90%)	272 (6.59%)
Unemployed, not looking for work	394 (14.06%)	523 (15.92%)	757 (17.34%)	623 (15.09%)
Gross annual household income				
Below median	1,884 (63.14%)	1,788 (51.17%)	2,156 (46.64%)	1,775 (40.29%)
Above median	1,100 (36.86%)	1,706 (48.83%)	2,467 (53.36%)	2,631 (59.71%)
Civic engagement				
Average-High	927 (39.33%)	1,176 (43.73%)	1,577 (43.73%)	1,368 (38.61%)
Low (Bottom quartile)	1,430 (60.67%)	1,513 (56.27%)	2,029 (56.27%)	2,175 (61.39%)
Community engagement				
Average-High	1,683 (72.17%)	1,926 (72.27%)	2,401 (67.05%)	2,225 (62.91%)
Low (Bottom Quartile)	649 (27.83%)	739 (27.73%)	1,180 (32.95%)	1,312 (37.09%)
Altruism				
Average-High	1,351 (56.93%)	1,455 (53.93%)	1,872 (51.83%)	1,512 (42.48%)
Low (Bottom quartile)	1,022 (43.07%)	1,243 (46.07%)	1,740 (48.17%)	2,047 (57.52%)
Cultural practices				
Average-High	1,202 (50.74%)	1,480 (54.92%)	1,910 (52.86%)	1,817 (51.04%)
Low (Bottom quartile)	1,167 (49.26%)	1,215 (45.08%)	1,703 (47.14%)	1,743 (48.96%)
Neighbourhood safety				
Average-High	1,114 (53.76%)	1,303 (55.47%)	1,934 (60.93%)	1,971 (62.89%)
Low (Bottom quartile)	958 (46.24%)	1,046 (44.53%)	1,240 (39.07%)	1,163 (37.11%)
Neighbourhood social cohesion				
Average-High	1,362 (57.81%)	1,629 (60.74%)	2,153 (59.91%)	2,188 (61.53%)
Low (Bottom quartile)	994 (42.19%)	1,053 (39.26%)	1,441 (40.09%)	1,368 (38.47%)
Neighbourhood atmosphere				
Average-High	1,161 (50.92%)	1,291 (50.37%)	1,753 (50.81%)	1,696 (49.53%)
Low (Bottom quartile)	1,119 (49.08%)	1,272 (49.63%)	1,697 (49.19%)	1,728 (50.47%)
Remoteness				
Major cities	2,125 (71.21%)	2,375 (67.99%)	3,247 (70.24%)	3,016 (68.48%)
Inner regional	505 (16.92%)	684 (19.58%)	864 (18.69%)	894 (20.30%)
Outer regional- very remote	354 (11.86%)	434 (12.42%)	512 (11.08%)	494 (11.22%)
SEIFA quintiles				
Low 1	595 (19.94%)	706 (20.21%)	961 (20.79%)	835 (18.95%)
2	575 (19.27%)	738 (21.12%)	931 (20.14%)	973 (22.08%)
3	567 (19.00%)	728 (20.84%)	993 (21.48%)	875 (19.86%)
4	613 (20.54%)	693 (19.83%)	901 (19.49%)	908 (20.61%)
High 5	634 (21.25%)	629 (18.00%)	837 (18.11%)	815 (18.50%)

Percentages may not equal 100 due to missing values. Further information about missing values included in Supplementary File 2.

**Table 3:** Unweighted descriptive statistics for the older population (60+) stratified by wave.

Factors	Wave			
	Wave 6	Wave 10	Wave 14	Wave 18
	*n* (%) (*N* = 2,873)	*n* (%) (*N* = 3,181)	*n* (%) (*N* = 4,424)	*n* (%) (*N* = 4,816)
Age				
60–64	753 (26.21%)	929 (29.20%)	1,218 (27.53%)	1,289 (26.76%)
65–69	651 (22.66%)	725 (22.79%)	1,091 (24.66%)	1,079 (22.40%)
70–74	518 (18.03%)	537 (16.88%)	778 (17.59%)	976 (20.27%)
75–79	472 (16.43%)	439 (13.80%)	572 (12.93%)	661 (13.73%)
80–84	308 (10.72%)	348 (10.94%)	425 (9.61%)	406 (8.43%)
85–89	126 (4.39%)	160 (5.03%)	242 (5.47%)	282 (5.86%)
90+	45 (1.57%)	43 (1.35%)	98 (2.22%)	123 (2.55%)
Gender				
Male	1,317 (45.84%)	1,485 (46.68%)	2,065 (46.68%)	2,231 (46.32%)
Female	1,556 (54.16%)	1,696 (53.32%)	2,359 (53.32%)	2,585 (53.68%)
Ethnicity				
Australian, non-indigenous	1,949 (70.92%)	2,077 (68.46%)	2,883 (68.20%)	3,168 (69.08%)
Australian, indigenous	26 (0.95%)	32 (1.05%)	38 (0.90%)	51 (1.11%)
Main English-speaking country born	391 (14.23%)	463 (15.26%)	649 (15.35%)	663 (14.46%)
Others	382 (13.90%)	462 (15.23%)	657 (15.54%)	704 (15.35%)
Marital status				
Legally married or de facto	1,739 (63.26%)	1,939 (63.87%)	2,756 (65.18%)	2,978 (64.95%)
Separated or divorced	290 (10.55%)	372 (12.25%)	542 (12.82%)	647 (14.11%)
Widowed	613 (22.30%)	602 (19.83%)	748 (17.69%)	744 (16.23%)
Never married and not de facto	107 (3.89%)	123 (4.05%)	182 (4.30%)	216 (4.71%)
Level of educational obtainment				
Tertiary level educated	332 (12.09%)	466 (15.37%)	778 (18.44%)	980 (21.40%)
Trade certificate	698 (25.42%)	807 (26.62%)	1,247 (29.55%)	1,468 (32.05%)
High school certificate	185 (6.74%)	237 (7.82%)	319 (7.56%)	364 (7.95%)
Did not finish high school	1,531 (55.75%)	1,522 (50.20%)	1,876 (44.45%)	1,768 (38.60%)
Self-assessed health				
Excellent	98 (4.00%)	104 (3.74%)	156 (4.02%)	165 (3.90%)
Very good	603 (24.62%)	714 (25.69%)	1,043 (26.89%)	1,122 (26.49%)
Good	956 (39.04%)	1,097 (39.47%)	1,502 (38.72%)	1,701 (40.17%)
Fair	634 (25.89%)	699 (25.15%)	928 (23.92%)	986 (23.28%)
Poor	158 (6.45%)	165 (5.94%)	250 (6.44%)	261 (6.16%)
Number of people in dwelling				
Multi-person household	2,038 (70.94%)	2,308 (72.56%)	3,246 (73.37%)	3,561 (73.94%)
Lone person household	835 (29.06%)	873 (27.44%)	1,178 (26.63%)	1,255 (26.06%)
Working status				
Employed	505 (18.37%)	666 (21.94%)	1,013 (23.95%)	1,115 (24.31%)
Unemployed, looking for work	14 (0.51%)	15 (0.49%)	30 (0.71%)	36 (0.78%)
Unemployed, not looking for work	2,230 (81.12%)	2,355 (77.57%)	3,186 (75.34%)	3,436 (74.91%)
Gross annual household income				
Below median	2,551 (88.79%)	2,608 (81.99%)	3,310 (74.82%)	3,406 (70.72%)
Above median	322 (11.21%)	573 (18.01%)	1,114 (25.18%)	1,410 (29.28%)
Civic engagement				
Average-High	1,300 (52.80%)	1,598 (58.32%)	2,171 (56.83%)	2,230 (53.10%)
Low (Bottom quartile)	1,162 (47.20%)	1,142 (41.68%)	1,649 (43.17%)	1,970 (46.90%)
Community engagement				
Average-High	1,764 (73.68%)	2,021 (75.92%)	2,776 (75.13%)	3,007 (73.36%)
Low (Bottom quartile)	630 (26.32%)	641 (24.08%)	919 (24.87%)	1,092 (26.64%)
Altruism				
Average-High	1,944 (78.14%)	2,187 (79.10%)	2,937 (76.17%)	3,025 (71.58%)
Low (Bottom quartile)	544 (21.86%)	578 (20.90%)	919 (23.83%)	1,201 (28.42%)
Cultural practices				
Average-High	1,645 (66.12%)	1,855 (67.14%)	2,458 (63.94%)	2,577 (61.05%)
Low (Bottom quartile)	843 (33.88%)	908 (32.86%)	1,386 (36.06%)	1,644 (38.95%)
Neighbourhood safety				
Average-High	1,662 (71.12%)	1,842 (72.18%)	2,713 (76.14%)	2,990 (77.62%)
Low (Bottom quartile)	675 (28.88%)	710 (27.82%)	850 (23.86%)	862 (22.38%)
Neighbourhood social cohesion				
Average-High	1,810 (75.39%)	2,074 (76.31%)	2,966 (78.13%)	3,280 (78.41%)
Low (Bottom quartile)	591 (24.61%)	644 (23.69%)	830 (21.87%)	903 (21.59%)
Neighbourhood atmosphere				
Average-High	1,565 (65.54%)	1,715 (64.62%)	2,350 (63.79%)	2,610 (63.72%)
Low (Bottom quartile)	823 (34.46%)	939 (35.38%)	1,334 (36.21%)	1,486 (36.28%)
Remoteness				
Major cities	1,716 (59.73%)	1,934 (60.80%)	2,702 (61.08%)	2,944 (61.13%)
Inner regional	750 (26.11%)	806 (25.34%)	1,120 (25.32%)	1,231 (25.56%)
Outer regional- very remote	407 (14.17%)	441 (13.86%)	602 (13.61%)	641 (13.31%)
SEIFA quintiles				
Low 1	734 (25.55%)	757 (23.80%)	1,055 (23.85%)	1,108 (23.01%)
2	592 (20.61%)	642 (20.18%)	923 (20.86%)	994 (20.64%)
3	581 (20.22%)	647 (20.34%)	829 (18.74%)	894 (18.56%)
4	476 (16.57%)	572 (17.98%)	805 (18.20%)	880 (18.27%)
High 5	490 (17.06%)	563 (17.70%)	812 (18.35%)	940 (19.52%)

Percentages may not equal 100 due to missing values. Further information about missing values included in Supplementary File 2.

### 3.2 Regression analysis

#### 3.2.1 Community participation determinants

**Figure 1:**
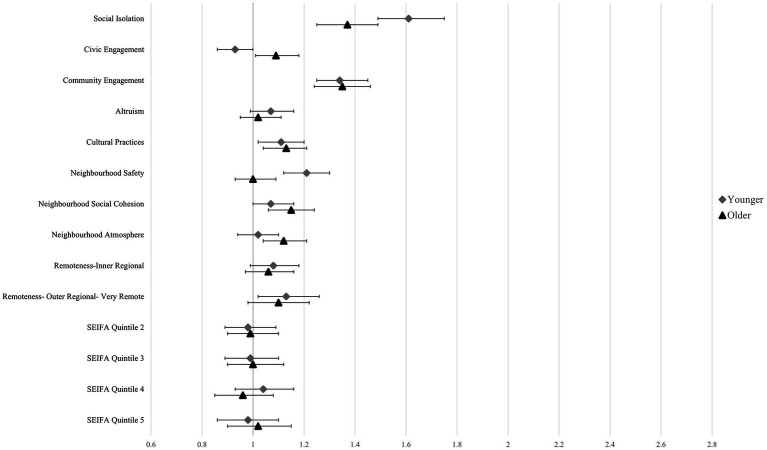
Regression plot of loneliness showing risk ratios and 95% confidence intervals. Reference categories were omitted.

**Figure 2:**
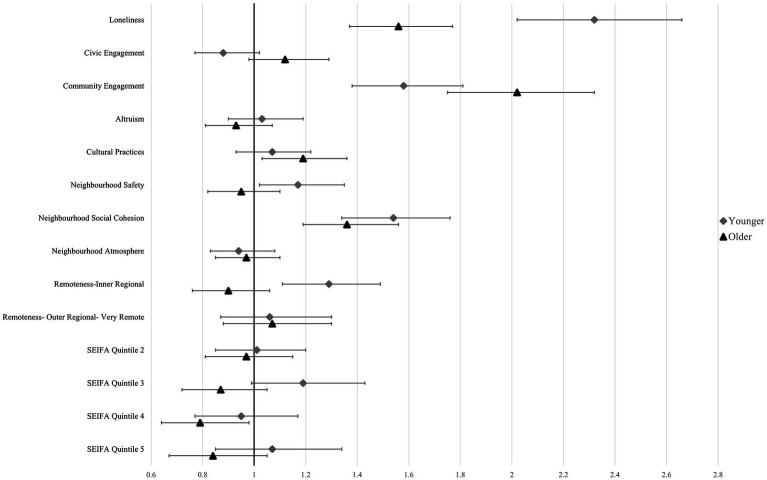
Regression plot of social isolation showing risk ratios and 95% confidence intervals. Reference categories were omitted.

Low altruism was not significantly associated with either outcome in either population, while civic engagement was only significantly associated with loneliness in the older population (RR:1.09, 95% CI:1.01–1.18). Low participation in cultural practices was significantly associated with an increased risk of loneliness in younger people (RR: 1.11, 95% CI:1.02–1.20) and older people (RR:1.13, 95% CI 1.04–1.21), and with a significantly increased risk of SI in older people (RR:1.19, 95% CI:1.03–1.36), but not in younger people. Low community engagement was strongly and significantly associated with an increased risk of loneliness and SI in both populations, where older people were more than twice as likely to experience SI if they had low community engagement scores (RR: 2.02, 95% CI: 1.75–2.32) and younger people were 1.5 times more likely to experience SI (RR:1.58, 95% CI: 1.38–1.81) (Table 4, Figures 1, 2).

**Table 4:** Poisson regression of loneliness and social isolation displaying risk ratios.

Variables	Loneliness		Social isolation	
Population	Younger (18–30)	Older (60+)	Younger (18–30)	Older (60+)
	RR (95% CI)	RR (95% CI)	RR (95% CI)	RR (95% CI)
Number of observations	8,215	9,876	8,220	9,875
Intercept	0.07 (0.06–0.09)***	0.07 (0.05–0.10)***	0.02 (0.01–0.03)***	0.05 (0.03–0.09)***
Loneliness				
Not lonely	-	-	Ref	Ref
Lonely	-	-	2.32 (2.02–2.66)***	1.56 (1.37–1.77)***
Social isolation				
Not isolated	Ref	Ref	-	-
Isolated	1.61 (1.49–1.75)***	1.37 (1.25–1.49)***	-	-
Time (Wave)	**1.01 (1.00–1.02)**	1.00 (0.99–1.01)	**1.01 (1.00–1.03)**	1.00 (0.98–1.01)
Community participation variables				
Civic engagement				
Average-High	Ref	Ref	Ref	Ref
Low (Bottom quartile)	0.93 (0.86–1.00)	1.09 (1.01–1.18)*	0.88 (0.77–1.02)	1.12 (0.98–1.29)
Community engagement				
Average-High	Ref	Ref	Ref	Ref
Low (Bottom quartile)	1.34 (1.25–1.45)***	1.35 (1.24–1.46)***	1.58 (1.38–1.81)***	2.02 (1.75–2.32)***
Altruism				
Average-High	Ref	Ref	Ref	Ref
Low (Bottom quartile)	1.07 (0.99–1.16)	1.02 (0.95–1.11)	1.03 (0.90–1.19)	0.93 (0.81–1.07)
Cultural practices				
Average-High	Ref	Ref	Ref	Ref
Low (Bottom quartile)	1.11 (1.02–1.20)*	1.13 (1.04–1.21)**	1.07 (0.93–1.22)	1.19 (1.03–1.36)*
Neighbourhood variables				
Neighbourhood safety				
Average-High	Ref	Ref	Ref	Ref
Low (Bottom quartile)	1.21 (1.12–1.30)***	1.00 (0.93–1.09)	1.17 (1.02–1.35)*	0.95 (0.82–1.10)
Neighbourhood social cohesion				
Average-High	Ref	Ref	Ref	Ref
Low (Bottom quartile)	**1.07 (1.00–1.16)**	1.15 (1.06–1.24)***	1.54 (1.34–1.76)***	1.36 (1.19–1.56)***
Neighbourhood atmosphere				
Average-High	Ref	Ref	Ref	Ref
Low (Bottom quartile)	1.02 (0.94–1.10)	1.12 (1.04–1.21)**	0.94 (0.83–1.08)	0.97 (0.85–1.10)
Remoteness				
Major cities	Ref	Ref	Ref	Ref
Inner regional	1.08 (0.99–1.18)	1.06 (0.97–1.16)	1.29 (1.11–1.49)***	0.90 (0.76–1.06)
Outer regional-very remote	1.13 (1.02–1.26)*	1.10 (0.98–1.22)	1.06 (0.87–1.30)	1.07 (0.88–1.30)
SEIFA quintiles				
Low 1	Ref	Ref	Ref	Ref
2	0.98 (0.89–1.09)	0.99 (0.90–1.10)	1.01 (0.85–1.20)	0.97 (0.81–1.15)
3	0.99 (0.89–1.10)	1.00 (0.90–1.12)	1.19 (0.99–1.43)	0.87 (0.72–1.05)
4	1.04 (0.93–1.16)	0.96 (0.85–1.08)	0.95 (0.77–1.17)	0.79 (0.64–0.98)*
High 5	0.98 (0.86–1.10)	1.02 (0.90–1.15)	1.07 (0.85–1.34)	0.84 (0.67–1.05)

*** *p* < 0.001, ** *p* < 0.01, * *p* < 0.05.

All models adjusted for the following individual and interpersonal level factors: age, gender, ethnicity, marital status, level of educational obtainment, self-assessed health, number of people in dwelling, working status, and gross annual household income.

Significant results are bolded.

#### 3.2.2 Neighbourhood determinants

SEIFA quintile was associated with the risk of SI in the older population where being in the second-most affluent SEFIA quintile was significantly associated with a 21% decrease in SI. Low perceived neighbourhood safety significantly increased the risk of loneliness (RR: 1.21, 95% CI: 1.12–1.30) and SI (RR: 1.17, 95% CI 1.02–1.35) in younger adults, however no association was found for either outcome variable in older people. Low neighbourhood social cohesion was significantly associated with SI in younger (RR: 1.54, 95% CI: 1.34–1.76) and older people (RR: 1.36, 95% CI: 1.19–1.56). There was a significant effect for loneliness in older people (RR: 1.15, 95% CI: 1.06–1.24), however, no significant effect was seen in the younger population for loneliness (Table 4, Figures 1, 2).

#### 3.2.3 Individual and interpersonal determinants

In the regression models, among both younger and older participants, being socially isolated strongly predicted loneliness and vice versa; being lonely was strongly and significantly prospectively associated with SI (Table 4).

Adjusted for all other variables, being female was significantly associated with a higher risk of loneliness and a lower risk of SI in both populations. Poorer self-assessed health was strongly and significantly associated with an increased risk of loneliness and SI in both populations. Being employed was associated with a decreased risk of loneliness and SI in younger people. Living in a lone person household was associated with an increased risk of loneliness in older people but was not significant in younger people. Further details may be found in Supplementary File 5.

## 4 Discussion

This is the first study to longitudinally identify such an extensive range of community-level determinants of loneliness and SI, comparing their effects among younger and older adults in the Australian context. Our findings support the utility of the social ecological model, demonstrating community level factors which affect the way that younger and older adults experience loneliness and SI. Community engagement emerged as the most influential community-level factor for both outcomes in younger and older adults, while social cohesion was also shown to reduce loneliness and SI in both populations. Additionally, there were several factors which were age specific, such as neighbourhood safety, neighbourhood atmosphere and cultural practices, highlighting important nuances in outcomes across generations. Our findings also confirm a strong relationship between loneliness and SI.

The importance of community engagement provides a strong rationale for community-level interventions to prevent and reduce loneliness and SI. This importance was particularly pronounced for the older adults’ experience of SI when compared to younger adults (RR: 2.02 vs. 1.58). This differential impact may be attributed to younger adults’ access to institutionalised pathways for social connection such as those found through employment and educational settings, a finding supported by our individual-level results where employment demonstrated protective effects against loneliness and SI in the younger cohort. For older adults, specific interventions to increase community engagement may be effective. Previous volunteer befriending interventions have been found to be beneficial in older populations, perhaps because they operationalise community engagement (26, 27). Interventions which allow older people to volunteer as well as be recipients of the programs may be even more beneficial for community loneliness reduction because of an additional effect of altruism (28).

Our results provide robust evidence for investigating loneliness and SI jointly due to their mutual influence, supporting previous work (2, 8). At the individual level, our results are also in agreeance with previously published literature, whereby gender was shown to have universal relevance in determining loneliness and SI (29). We also replicate results showing that marriage and household size are not consistent predictors across both age groups (29). Despite some individual-level factors, such as gender or self-assessed, health having a stronger effect on loneliness and SI, they are less amendable to change, highlighting the need for action on every level of the social ecological model to appropriately address loneliness and SI.

Our findings underscore the complex interplay between individual, interpersonal, community and societal-level factors in shaping experiences of loneliness and SI, further demonstrating how factors at different ecological levels may interact dynamically, creating both vulnerabilities and opportunities for intervention that would be missed by single-level analyses. Similarly, Holt-Lunstad (30) emphasises that social disconnection emerges from interactions between personal vulnerabilities and environmental contexts, with neither alone fully explaining outcomes. This multi-level interaction may explain why certain community factors, such as neighbourhood safety, have differential impacts across age groups, reflecting not just varying environmental needs but also age-related psychological resources and coping mechanisms. These interactions highlight the need for interventions that simultaneously address multiple levels of the social ecological model, bridging individual characteristics with community resources in ways that create meaningful connections.

The literature supporting the effect of neighbourhood social cohesion among older people is well developed (6, 31), but is less robust among younger people (32). Our study demonstrates that social cohesion can also impact loneliness and SI in younger people, albeit a weaker association than for community engagement. Our results indicate that increasing social cohesion in the community would benefit both the younger and older populations. Previous research suggests that this may be achieved through countering social stereotypes and providing opportunities for positive interactions (33).

Neighbourhood safety, neighbourhood atmosphere and cultural practices affected each age group differently. The cultural practice variable elicited an effect on younger adults’ loneliness but not SI, and older adults’ loneliness and SI, suggesting different causal pathways which may require different intervention approaches. Younger people are less likely to identify as religious than older people, and therefore, unlike older people, are less inclined to experience going to religious places as a social practice (34). Therefore, attending religious services has differential effects for each age cohort. Younger people who do engage in religious services however, draw a much stronger sense of identity from these practices, lowering their potential for loneliness when compared to those who do not engage in religious services (35).

Low perceived neighbourhood safety was seen to predict loneliness in younger and older people in our study, consistent with previous research (36). Approaches that improve the neighbourhood safety in communities with a high proportion of younger or older adults may help reduce loneliness. Previous studies have shown a relationship between social cohesion and neighbourhood safety, allowing for considerations of neighbourhood safety to be incorporated into population approaches for improving neighbourhood social cohesion, rather than as standalone interventions (37).

Contrary to previous studies (20), we did not find an association between SEIFA and loneliness and SI, except for a weak protective effect over SI in older adults in the second-highest SEIFA quintile. We hypothesise that community-level factors may mitigate the effect that SEIFA has on the outcomes, demonstrating that SEIFA may not be a good indicator of the risk of loneliness or SI in a population. Lower socio-economic areas typically have lower offerings of activities, which may hinder peoples’ ability to connect (38).

A strength of this study is our longitudinal design, using the HILDA dataset. HILDA has a robust study design and covers a wide range of variables across the individual, interpersonal and community levels allowing investigation of factors at the community-level, while controlling for factors from the other levels, addressing a research gap identified in a previous review (6). However, the HILDA data does not assess other community-level factors and potential confounders, such as the effect of personality, open green space and transport availability, which have also been found to influence loneliness and SI. While our longitudinal design helps reduce the impact of time-invariant unmeasured confounders, time-varying unmeasured factors could still influence our findings, therefore further investigation through data linkage studies would be beneficial (39).

A limitation of this study is the reliance on self-reported responses as an indirect proxy for determining community-level variables, which may introduce differential measurement bias where those who are lonely or isolated may overstate or understate the effects of community variables (40). However, self-reported data can be a reliable measurement tool in neighbourhood settings particularly those that are answered on a large-scale, which have been shown to have high internal-consistency and re-test reliability (41). Further, we constructed covariates by collapsing several questions under one concept category (e.g., cultural practice, neighbourhood atmosphere) to minimise the number of factors, limiting our ability to identify which aspect is more influential.

Our sample was drawn from a nationally representative sample in the Australian context; however, some caution should be employed when generalising our findings. The HILDA sample only includes private dwellings, therefore those who are in unstable housing and those who reside in institutions, including those living in defence force dwellings and aged-care homes are not captured in this study, representing a limitation, particularly in the younger and older adults who are more likely to be in these populations. Additionally, when generalising these findings outside of the Australian context, specific cultural considerations and context specific factors must be considered. Further research is needed to elucidate the extent of these limitations.

### 4.1 Implications for research, policy and practice

Given this study used research-led quantitative conceptualisation of community-level determinants, it will be important to further examine how Australian younger and older people understand loneliness and SI in their lives and how community-level factors may influence their experiences using qualitative methods. In addition, leaders in the fields of loneliness and SI research have called for a systems approach as a priority for future research (42), as is exemplified by our results. Evaluations of future interventions which account for all levels of influence should be undertaken to examine whether there is added benefit of using the social ecological framework when compared to an individualised approach. Future research should adopt a life-course approach ensuring prevention can occur at the primordial level rather than targeting only those at most risk or those already suffering from loneliness and/or SI. Interventions that prioritise creating healthy connections and cultural identity within a community, through formation of community interest groups and clubs; or evaluations of social policy changes, such the implementation of free community events and park upgrades, are necessary. Specific policy recommendations include ensuring the continuity of community groups through targeted and sustainable funding, investing in spaces with community-led design which would include parks, community centres and other public third places. Additionally, implementing free or low-cost inter-generational events may help to improve social cohesion across younger and older populations.

## 5 Conclusion

By identifying and comparing the effects of an extensive range of community-level determinants of loneliness and SI among younger and older adults in the Australian context, our findings demonstrate community level factors which affect the way that these populations experience loneliness and SI. The findings highlight age-specific nuances, such as those for neighbourhood safety, neighbourhood atmosphere and cultural practices, which provide evidence for the need to account for all levels of the social ecological model in determining appropriate interventions and solutions. Our study highlights the importance of using a social ecological framework when designing public health programs to address loneliness and SI in younger and older people as there needs to be consideration of the individual, interpersonal, community and societal factors in tandem to effectively prevent and reduce loneliness and SI.

## Data Availability

The data analyzed in this study is subject to the following licenses/restrictions: this paper uses unit record data from Household, Income and Labour Dynamics in Australia Survey [HILDA] conducted by the Australian Government Department of Social Services (DSS). The findings and views reported in this paper, however, are those of the author[s] and should not be attributed to the Australian Government, DSS, or any of DSS’ contractors or partners, doi: 10.26193/PI5LPJ. Requests to access these datasets should be directed to Australian Government Department of Social Services (DSS), doi: 10.26193/PI5LPJ.
