# Supplementary material for: Community-level determinants of loneliness and social isolation: a population-based cohort study across younger and older adults
Source: Front Public Health. 2025 May 15;13:1526166. doi: 10.3389/fpubh.2025.1526166 (PMC12119266; doi:10.3389/fpubh.2025.1526166)
Supplement: Supplementary file 1 [file Supplementary_file_1.docx]

**Supplementary File 1.**

Details and construction for each variable used in the analysis.

| **Variable** | **Question on survey** | **Description** | **Survey component** | **Variable name in HILDA dataset** | **Variable operationalisation for analysis** | **Categories** | **Reference** |
| --- | --- | --- | --- | --- | --- | --- | --- |
| **Identifier Variable** |  |  |  |  |  |  |  |
| Person ID | NQ-Recorded by interviewer | Person ID | HF | _xwaveid |  |  |  |
| **Time Variable** |  |  |  |  |  |  |  |
| Wave | NQ- Recorded by interviewer | Time | HF | Wave prefix | Categorised according to time point. | 6=2006  10=2010  14=2014  18=2018 |  |
| **Outcome Variables** |  |  |  |  |  |  |  |
| Loneliness | - People don’t come to visit me as often as I would like - I often need help from other people but can’t get it - I often feel very lonely | Loneliness experienced by participants | SCQ | _lssupv  _lssupnh  _lssupvl | Individual responses for each question were recorded on Likert scales of 1-7 with 1 representing strongly disagree and 7 representing strongly agree. We classified people who were lonely to have a median score of ≥4. | 1=lonely  2=not lonely | Scale used by Lim, Manera et al. (2023) |
| Social Isolation | - There is someone who can always cheer me up when I’m down - I enjoy the time I spend with the people who are important to me - When somethings on my mind, just talking with the people I know can make me feel better - When I need someone to help me out, I can usually find someone | Social isolation experienced by participants | SCQ | _lssupcd  _lssupi  _lssuptp  _lssupsh | Individual responses for each question were recorded on Likert scales of 1-7 with 1 representing strongly disagree and 7 representing strongly agree. We classified people who were socially isolated to have a median score of ≤4. | 1=isolated  2=not isolated | Scale used by Lim, Manera et al. (2023) |
| **Individual and Interpersonal Variables** |  |  |  |  |  |  |  |
| Age | - Year of Birth | Age of participant in years at last birthday | PQ | _hgage | Categorised into intervals of 5 years in the older population.  Categorised into intervals of 4 years in the younger population. |  |  |
| Gender | NQ- Recorded by interviewer | Gender of participant (limited to male or female) | HF | _hgsex | Categories provided by the HILDA data custodians, which were male and female. There was no ‘other’ option provided in the HILDA survey. | 1=Male  2=Female |  |
| Ethnicity | - In which country were you born? - Are you of Aboriginal or Torres Strait Islander origin? | Place of birth including Aboriginal and Torres Strait Islander status | PQ | _anbcob  _anatsi | Categories provided by the HILDA data custodians. An extra category was added for Australian Indigenous status for those who identified as Aboriginal or Torres Strait Islander.  Main English-speaking countries are defined as: United Kingdom, New Zealand, Canada, USA, Ireland and South Africa. | 1=Australian, non-Indigenous  2=Australian, Indigenous  3=Main English speaking country born  4=Others |  |
| Marital Status | - Current marital status - Currently living with someone in a relationship | Marital status of participant | PQ | _mrcurr | Categories provided by the HILDA data custodians. | 1=legally married  2=de facto  3=separated  4=divorced  5=widowed  6= never married and not de facto |  |
| Level of Educational Obtainment | - Age left school - Highest education level achieved. - Highest year of school completed (excludes equivalents obtained post-school)/currently attending - Number of qualifications obtained since leaving school | Highest level of educational obtainment at the time of the survey | PQ | _edagels  _edhigh1 | Highest education level achieved (_edhigh1) was collapsed into 4 categories denoting tertiary level education (including postgrad, grad diploma and bachelors or honours), trade certificate (including those completing an adv diploma and a cert III or IV), high school certificate (those who completed year 12), and did not finish high school (those who achieved year 11 or below). A fifth category was included for those still in school, given our young sample, which was determined using ‘_edage’. | 1=Tertiary level educated  2=Trade certificate  3=High school certificate  4=Did not finish high school  5=Still in school |  |
| Self-Assessed Health | - In general, would you say your health is: excellent, very good, good, fair, poor | Participant’s self-rated health | SCQ | _gh1 | Categories provided by the HILDA data custodians. | 1=Excellent  2=Very Good  3=Good  4=Fair  5=Poor |  |
| Number of People in Dwelling | NQ- Recorded by the interviewer | Number of in-scope persons in the dwelling | HF | _hhpers | Dichotomised according to number of in scope people in the household with lone-person households and those with multiple people. | 1=lone-person household  2=multi-person household |  |
| Working Status | - Current employment status - Done any of following in last 4 weeks   - Answered an advertisement for a job   - Advertised or tendered for work   - Checked or registered with an employment agency   - Checked factory noticeboards, or used the touchscreens at Centrelink offices   - Contacted friends/relatives   - Been registered with Centrelink as a jobseeker   - Written, phoned or applied in person to an employer for work - If found a job, could have started work last week - Looked for work in last 4 weeks - Reasons could not have started work last week - If offered suitable job, start work in next four weeks | Current labour force status | PQ | _esdtl | Categories collapsed where ‘employed’ included those working full-time and part-time and those whose usula working hours were unknown, ‘unemployed, looking for work’ included those looking for full-time and part-time work, and ‘unemployed, not looking for work’ included those not in the labour force, whether they were marginally attached or not, where marginal attachment includes those who may want to work but are not actively pursuing employment. | 1=Employed  2=Unemployed, looking for work  3=Unemployed, not looking for work |  |
| Gross Annual Household Income | For each member of the household:   - Financial year Australian public transfers (inc family benefits) ($) - Financial year foreign pensions ($) - Financial year Other regular public including scholarships ($) - Financial year regular private income ($) Negative values - Financial year regular private income ($) Positive values | Gross regular household income for participant | PQ | _hifefp  _hifefn | Dichotomised using the median household income of $92,872AUD a year as per Australian Bureau of Statistics. Imputed values were used, and all values are presented in AUD. | 1=Above Median  2=Below Median | <https://www.abs.gov.au/statistics/economy/finance/household-income-and-wealth-australia/latest-release> |
| **Community Participation Variables** |  |  |  |  |  |  |  |
| Civic Engagement | - In general, how often do you do the following things:   - Get involved in activities for a union, political party, or group that is for or against something   - Encourage others to get involved with a group thats trying to make a difference in the community   - Talk about current affairs with friends, family or neighbours   - Get in touch with a local politician or councillor about issues that concern me | The extent to which participants engage in activities or groups that aim to influence the community and society at large. | SCQ | _lsnwpol  _lsnwinv  _lsnwtca  _lsnwpi | Responses were recorded on a Likert scale where 1 was ‘never’ and 6 was ‘all the time’. Scores were totalled and the mean of the scores was determined. Using a data driven approach, quartiles determined the cut point, where the bottom quartile was categorised as having low civic engagement compared to those with responses in the other quartiles. The cut-point was ≤2. | 1=Lowest quartile  2=Other quartiles |  |
| Community Engagement | - In general, how often do you do the following things:   - Have telephone, email or mail contact with friends or relatives not living with you   - Chat with your neighbours   - Make time to keep in touch with friends   - See members of my extended family (or relatives not living with me) in person - How often get together socially with friends/relatives not living with you? | How often participants engage in social activities with friends and relatives. | SCQ | _lsnwcon  _lsnwcht  _lsnwkit  _lsnwexf  _lssocal | Responses were recorded on a Likert scale where 1 was ‘never’ and 6 was ‘all the time’ for the first question set.  ‘How often get together socially with friends/relatives not living with you?’ was asked on a 7 point scale, from 1-every day to 7-less often than once every 3 months. This was reverse coded so the higher score denoted a more frequent engagement.  Scores were totalled and the mean of the scores was determined. Using a data driven approach, quartiles determined the cut point, where the bottom quartile was categorised as having low engagement compared to those with responses in the other quartiles. The cut-point was ≤4.27. | 1= Top three quartiles  2= Lowest quartile |  |
| Altruism | - In general, how often do you do the following things:   - Volunteer your spare time to work on boards or organising committees of clubs, community groups or other non-profit organisations   - Give money to charity if asked | The extent to which participants actively contribute to the community or non-profit organisations | SCQ | _lsnwvol  _lsnwmc | Responses were recorded on a Likert scale where 1 was ‘never’ and 6 was ‘all the time’. Scores were totalled and the mean of the scores was determined. Using a data driven approach, quartiles determined the cut point, where the bottom quartile was categorised as having low altruism compared to those with responses in the other quartiles. The cut-point was ≤2. | 1= Top three quartiles  2= Lowest quartile |  |
| Cultural Practices | - In general, how often do you do the following things:   - Attend events that bring people together such as fetes, shows, festivals or other community events - Make time to attend services at a place of worship | Participants participation in events and spiritual practices that are characteristic of their community | SCQ | _lsnwce  _lsnwser | Responses were recorded on a Likert scale where 1 was ‘never’ and 6 was ‘all the time’. Scores were totalled and the mean of the scores was determined.. Using a data driven approach, quartiles determined the cut point, where the bottom quartile was categorised as having low cultural engagement compared to those with responses in the other quartiles. The cut-point was ≤2. | 1= Top three quartiles  2= Lowest quartile |  |
| **Neighbourhood Variables** |  |  |  |  |  |  |  |
| Neighbourhood Safety | - How common are the following things in your local neighbourhood?   - People being hostile and aggressive   - Teenagers hanging around on the streets   - Vandalism and deliberate damage to property   - Burglary and theft | Participants perception of safety in their local neighbourhood | SCQ | _lslaha  _lslats  _lslavd  _lslabt | Responses were recorded on a Likert scale where 1 was ‘never happens’ and 5 was ‘very common’. Scores were totalled and the mean of the scores was determined. Using a data driven approach, quartiles determined the cut point, where the bottom quartile was categorised as having low safety perceptions compared to those with responses in the other quartiles. The cut-point was ≤3.25. | 1= Top three quartiles  2= Lowest quartile |  |
| Neighbourhood Social Cohesion | - To what extent do you agree or disagree with the following statements about your neighbourhood?   - This is a close-knit neighbourhood   - People around here are willing to help their neighbours   - People in this neighbourhood can be trusted   - People in this neighbourhood generally do not get along - People in this neighbourhood generally do not share the same values | Participants perception of cohesion in their local neighbourhoods including their shared values. | SCQ | _lslackn  _lslawhn  _lslatr  _lslanga  _lslansv | Responses were recorded on a Likert scale where 1 was ‘strongly disagree’ and 7 was ‘strongly agree’. The negative questions (‘people in this neighbourhood generally do not get along’ and ‘people in this neighbourhood generally do not share the same values’) were reverse coded. Scores were totalled and the mean of the scores was determined.. Using a data driven approach, quartiles determined the cut point, where the bottom quartile was categorised as having low perceptions of social cohesion compared to those with responses in the other quartiles. The cut-point was ≤4. | 1= Top three quartiles  2= Lowest quartile | Scale first used by Sampson, Raudenbush et al. (1997) |
| Neighbourhood Atmosphere | - How common are the following things in your local neighbourhood?   - Traffic noise   - Noise from airplanes, trains or industry   - Homes and gardens in bad condition | Participants perception of the neighbourhood environment including noise levels and the aesthetics. | SCQ | _lslatn  _lslaat  _lslahg | Responses were recorded on a Likert scale where 1 was ‘never happens’ and 5 was ‘very common’. Scores were totalled and the mean of the scores was determined.. Using a data driven approach, quartiles determined the cut point, where the bottom quartile was categorised as having negative perceptions of the neighbourhood atmosphere compared to those with responses in the other quartiles. The cut-point was ≤2.5. | 1= Top three quartiles  2= Lowest quartile |  |
| Remoteness | - - NQ- Recorded by interviewer based on the participants primary address. | Australian Statistical Geography Standard, 2011 Remoteness Areas | HF | _hhsra | Categories provided by the HILDA data custodians, with outer regional to very remote categories collapsed due to small sample size. Those with no usual address and offshore addresses were excluded. | 1=Major cities  2=Inner regional  3=Outer regional-very remote | (Australian Bureau of Statistics 2023) |
| SEIFA Quintiles | NQ-Recorded by interviewer | SEIFA 2011 Index of relative socio-economic advantage/disadvantage | HF | _hhsad | Provided by the HILDA data custodians as a continuous variable, and this was categorised into quintiles, with 1 denoting the most disadvantaged and 5 representing the most advantaged. | 1=Lowest quintile  2  3  4  5=Highest quintile | (Australian Bureau of Statistics 2023) |

*Note: NQ- No question, SCQ- Self-Complete Questionnaire, PQ- Person Questionnaire, HQ- Household Questionnaire, HF-Household Form*

Australian Bureau of Statistics. (2023). "Census of Population and Housing: Socio-Economic Indexes for Areas (SEIFA), Australia, 2011." Retrieved 23 November 2023, from <https://www.abs.gov.au/AUSSTATS/abs@.nsf/allprimarymainfeatures/8C5F5BB699A0921CCA258259000BA619?opendocument>=.

Australian Bureau of Statistics. (2023). "Remoteness Structure." Retrieved 23 November 2023, from <https://www.abs.gov.au/AUSSTATS/abs@.nsf/Lookup/1270.0.55.005Main+Features1July%202016?OpenDocument>=.

Lim, M. H., K. E. Manera, K. B. Owen, P. Phongsavan and B. J. Smith (2023). "The prevalence of chronic and episodic loneliness and social isolation from a longitudinal survey." Scientific Reports **13**(1): 12453.

Sampson, R. J., S. W. Raudenbush and F. Earls (1997). "Neighborhoods and violent crime: A multilevel study of collective efficacy." Science **277**(5328): 918-924.
