# Supplementary material for: Community-level determinants of loneliness and social isolation: a population-based cohort study across younger and older adults
Source: Front Public Health. 2025 May 15;13:1526166. doi: 10.3389/fpubh.2025.1526166 (PMC12119266; doi:10.3389/fpubh.2025.1526166)
Supplement: Supplementary file 2 [file Supplementary_file_2.docx]

**Supplementary File 2.**

Missing values investigations

**Supp Table 1**. Patterns of missing data due to non-participation stratified by population. SCQ=Self-completed questionnaire

|  | **Younger Population- Aged 18-30 n(%)** | | | | | **Older Population- Aged 60+ n(%)** | | | | |
| --- | --- | --- | --- | --- | --- | --- | --- | --- | --- | --- |
| Wave | **6** | **10** | **14** | **18** | **Total** | **6** | **10** | **14** | **18** | **Total** |
| Total Population | 2,984 | 3,494 | 4,623 | 4,406 | 15,507 | 2,873 | 3,181 | 4,424 | 4,816 | 15,294 |
| Not interviewed | 181 (6.1) | 209 (6.0) | 258 (5.6) | 277 (6.3) | 925 (6.0) | 124 (4.3) | 145 (4.6) | 195 (4.4) | 229 (4.8) | 693 (4.5) |
| No SCQ Returned | 410 (13.7) | 560 (16.0) | 710 (15.4) | 515 (11.7) | 2,195 (14.2) | 186 (6.5) | 225 (7.1) | 309 (7.0) | 276 (5.7) | 996 (6.5) |
| SCQ Returned | 2,393 (80.2) | 2,725 (78.0) | 3,655 (79.1) | 3,614 (82.0) | 12,387 (79.9) | 2,563 (89.2) | 2,811 (88.4) | 3,920 (88.6) | 4,311 (89.5) | 13,605 (89.0) |

**Supp Table 2.** Patterns of missing data due to non-participation for the total included population (18-30 & 60+)

| **Wave** | **6** | **10** | **14** | **18** | **Total** |
| --- | --- | --- | --- | --- | --- |
| Total Population | 5,857 | 6,675 | 9,047 | 9,222 | 30,801 |
| Not interviewed | 305 (5.2) | 354 (5.3) | 453 (5.0) | 506 (5.5) | 1,618 (5.3) |
| No SCQ Returned | 596 (10.2) | 785 (11.8) | 1,019 (11.3) | 791 (8.6) | 3,191 (10.4) |
| SCQ Returned | 4,956 (84.6) | 5,536 (82.9) | 7,575 (83.7) | 7,925 (85.9) | 25,992 (84.4) |

Further missing values investigations only take into account those who have returned a SCQ, which is required to be included in the regression analyses.

**Supp Table 3.** Missing values for each variable across waves 6,10,14 and 18 where the total population is 25,992, and included people 18-30 and 60+ years of age.

| **Variable** | **Missing Observations n(%)** | **Completed (Not Missing) Observations** |
| --- | --- | --- |
| Age | 0 (0.0) | 25,992 |
| Gender | 0 (0.0) | 25,992 |
| Ethnicity | 9 (0.0) | 25,983 |
| Marital Status | 2 (0.0) | 25,990 |
| Level of Educational Obtainment | 20 (0.1) | 25,972 |
| Self-assessed health | 478 (1.8) | 25,514 |
| Number of people in dwelling | 0 (0.0) | 25,992 |
| Working Status | 0 (0.0) | 25,992 |
| Gross Annual Household Income | 0 (0.0) | 25,992 |
| Civic Engagement | 575 (2.2) | 25,417 |
| Community Engagement | 1,027 (4.0) | 24,965 |
| Altruism | 415 (1.6) | 25,577 |
| Cultural Practices | 439 (1.7) | 25,553 |
| Neighbourhood Safety | 2,959 (11.4) | 23,033 |
| Neighbourhood Social Cohesion | 706 (2.7) | 25,286 |
| Neighbourhood Atmosphere | 1,453 (5.6) | 24,539 |
| Remoteness | 2 (0.0) | 25,990 |
| SEIFA Quintiles | 0 (0.0) | 25,992 |

**Supp Table 4**. Missing value patterns where ‘X’ denotes missing values with total number of observations following the pattern displayed in the totals row. Patterns with less than 100 observations were omitted and account for less than 5% of the total sample. 20494 observations were recorded with zero missing values. Analysis included people 18-30 and 60+.

| **Pattern** | **1** | **2** | **3** | **4** | **5** | **6** | **7** | **8** |
| --- | --- | --- | --- | --- | --- | --- | --- | --- |
| Age |  |  |  |  |  |  |  |  |
| Gender |  |  |  |  |  |  |  |  |
| Ethnicity |  |  |  |  |  |  |  |  |
| Marital Status |  |  |  |  |  |  |  |  |
| Level of Educational Obtainment |  |  |  |  |  |  |  |  |
| Self-assessed health |  |  |  |  | X |  |  |  |
| Number of people in dwelling |  |  |  |  |  |  |  |  |
| Working Status |  |  |  |  |  |  |  |  |
| Gross Annual Household Income |  |  |  |  |  |  |  |  |
| Civic Engagement |  |  |  |  |  |  | X | X |
| Community Engagement |  |  |  | X |  |  | X |  |
| Altruism |  |  |  |  |  |  | X |  |
| Cultural Practices |  |  |  |  |  |  | X |  |
| Neighbourhood Safety | X |  | X |  |  |  |  |  |
| Neighbourhood Social Cohesion |  |  |  |  |  | X |  |  |
| Neighbourhood Atmosphere |  | X | X |  |  |  |  |  |
| Remoteness |  |  |  |  |  |  |  |  |
| SEIFA Quintiles |  |  |  |  |  |  |  |  |
| **Total Observations**  **n(%)** | **1,909 (7.3)** | **506 (1.9)** | **505 (1.9)** | **427 (1.6)** | **334 (1.3)** | **259 (1.0)** | **117 (0.5)** | **111 (0.4)** |

**Supp Table 5.** Reasons for missingness across variables used to construct ‘neighbourhood safety’

| **Variable** | **Response** | **Wave n(%)** | | | | |
| --- | --- | --- | --- | --- | --- | --- |
|  |  | **6** | **10** | **14** | **18** | **Total** |
| People being hostile and aggressive | Multiple responses | 3 (0.1) | 3 (0.1) | 3 (0.0) | 2 (0.0) | 11 (0.0) |
|  | Refused/Not stated | 81 (1.6) | 53 (1.0) | 69 (0.9) | 82 (1.0) | 285 (1.1) |
|  | Don’t know | 134 (2.7) | 167 (3.0) | 235 (3.1) | 264 (3.3) | 800 (3.1) |
| Teenagers hanging around on the streets | Multiple responses | 4 (0.1) | 2 (0.0) | 4 (0.1) | 1 (0.0) | 11 (0.0) |
|  | Refused/Not stated | 81 (1.6) | 56 (1.0) | 63 (0.8) | 80 (1.0) | 280 (1.1) |
|  | Don’t know | 96 (1.9) | 106 (1.9) | 176 (2.3) | 223 (2.8) | 601 (2.3) |
| Vandalism and  deliberate damage to  property | Multiple responses | 2 (0.0) | 3 (0.1) | 3 (0.0) | 0 (0.0) | 8 (0.0) |
|  | Refused/Not stated | 73 (1.5) | 53 (1.0) | 61 (0.8) | 74 (0.9) | 261 (1.0) |
|  | Don’t know | 139 (2.8) | 165 (3.0) | 246 (3.2) | 309 (3.9) | 859 (3.3) |
| Burglary and theft | Multiple responses | 1 (0.0) | 3 (0.1) | 2 (0.0) | 0 (0.0) | 6 (0.0) |
|  | Refused/Not stated | 72 (1.5) | 52 (0.9) | 64 (0.8) | 78 (1.0) | 266 (1.0) |
|  | Don’t know | 369 (7.4) | 488 (8.8) | 636 (8.4) | 704 (8.9) | 2,197 (8.5) |
