# Supplementary material for: Community-level determinants of loneliness and social isolation: a population-based cohort study across younger and older adults
Source: Front Public Health. 2025 May 15;13:1526166. doi: 10.3389/fpubh.2025.1526166 (PMC12119266; doi:10.3389/fpubh.2025.1526166)
Supplement: Supplementary file 3 [file Supplementary_file_3.docx]

**Supplementary File 3.**

Sensitivity analyses using continuous/binary outcomes variables and with continuous/categorical covariates. Only full models for 18-30 year olds are shown.

**Supp Table 1.** *Model fit test for loneliness and social isolation models comparing* *continuous/binary outcomes variables and with continuous/categorical covariates using the Bayesian Information Criterion. Bolded row indicates best fit for each model.*

| Model | Number of Observations | | Degrees of freedom | BIC | $\boldsymbol{\Delta i-\Delta min}$ |
| --- | --- | --- | --- | --- | --- |
| Younger People | |  | | | |
| *Loneliness* | |  | | | |
| Continuous loneliness variable, continuous co-variates | 8215 | | 33 | 29023.90 | 19038.26 |
| Continuous loneliness variable, categorical co-variates | 8215 | | 38 | 29404.04 | 19418.40 |
| Categorical loneliness variable, continuous co-variates | **8215** | | **33** | **9985.64** | **0** |
| Categorical loneliness variable, categorical co-variates | 8215 | | 38 | 10146.17 | 160.53 |
| *Social Isolation* | |  | | | |
| Continuous social isolation variable, continuous co-variates | 8220 | | 33 | 31683.12 | 26409.67 |
| Continuous social isolation variable, categorical co-variates | 8220 | | 38 | 31793.02 | 26519.57 |
| Categorical social isolation variable, continuous co-variates | **8220** | | **33** | **5273.45** | **0** |
| Categorical social isolation variable, categorical co-variates | 8220 | | 38 | 5328.39 | 54.94 |
| Older People | |  | | | |
| *Loneliness* | |  | | | |
| Continuous loneliness variable, continuous co-variates | 9876 | | 32 | 35160.99 | 23677.65 |
| Continuous loneliness variable, categorical co-variates | 9876 | | 40 | 35699.96 | 24216.62 |
| Categorical loneliness variable, continuous co-variates | **9876** | | **32** | **11483.34** | **0** |
| Categorical loneliness variable, categorical co-variates | 9876 | | 40 | 11659.24 | 175.90 |
| *Social Isolation* | |  | | | |
| Continuous social isolation variable, continuous co-variates | 9875 | | 32 | 38779.91 | 32328.55 |
| Continuous social isolation variable, categorical co-variates | 9875 | | 40 | 38931.85 | 32480.49 |
| Categorical social isolation variable, continuous co-variates | **9875** | | **32** | **6451.36** | **0** |
| Categorical social isolation variable, categorical co-variates | 9875 | | 40 | 6591.52 | 140.16 |

**Supp. Table 2.** *Loneliness and social isolation in participants aged 18-30. Significant results are bolded. p values included where significant *p < 0.05; **p < 0.01; ***p < 0.001. Red text denotes values which for which significance changed when dichotomised in final models. Note: Reference levels provided for categorical models in brackets where relevant.*

| **Variables** | **Loneliness** | | **Social Isolation** | |
| --- | --- | --- | --- | --- |
|  | **Full Continuous** | **Full Categorical** | **Full Continuous** | **Full Categorical** |
|  | **RR (95% CI)** | **RR (95% CI)** | **RR (95% CI)** | **RR (95% CI)** |
| **Number of observations** | 5634 | 5634 | 5635 | 5635 |
| **Social Isolation**  (Not socially isolated) | **0.88 (0.87-0.89)***** | **1.61 (1.49-1.75)***** | - | - |
| **Loneliness**  (Not lonely) | - | - | **0.97 (0.96-0.97)***** | **2.32 (2.02-2.66)***** |
| ***Individual and Interpersonal Variables*** | | | | |
| **Age** | 1.00 (1.00-1.01) |  | 1.00 (1.00-1.00) |  |
| 18-21 |  | Ref |  | Ref |
| 22-24 |  | 1.07 (0.98-1.17) |  | 0.98 (0.83-1.15) |
| 25-27 |  | 1.06 (0.95-1.17) |  | 1.09 (0.91-1.31) |
| 28-30 |  | 1.07 (0.96-1.19) |  | 1.00 (0.83-1.21) |
| **Gender** | | | | |
| Male | Ref | Ref | Ref | Ref |
| Female | **1.14 (1.11-1.18)***** | **1.21 (1.13-1.31)***** | **1.04 (1.03-1.05)***** | **0.69 (0.61-0.79)***** |
| **Ethnicity** | | | | |
| Australian, non-Indigenous | Ref | Ref | Ref | Ref |
| Australian, Indigenous | 1.02 (0.95-1.09) | 1.00 (0.86-1.15) | 1.00 (0.97-1.02) | 1.20 (0.96-1.49) |
| Main English-speaking country born | **1.12 (1.04-1.20)**** | **1.24 (1.03-1.49)*** | 1.01 (0.99-1.03) | 1.09 (0.75-1.60) |
| Others | 1.03 (0.98-1.09) | 1.05 (0.90-1.23) | 1.00 (0.99-1.02) | 1.08 (0.81-1.44) |
| **Marital Status** | | | | |
| Legally married or de facto | Ref | Ref | Ref | Ref |
| Separated or divorced | 1.10 (0.97-1.24) | 1.25 (0.94-1.66) | 0.97 (0.92-1.02) | **1.60 (1.03-2.49)*** |
| Widowed | - | - | - | **-** |
| Never married and not de facto | **1.16 (1.12-1.20)***** | **1.36 (1.24-1.48)***** | **0.99 (0.98-1.00)**** | **1.24 (1.07-1.45)**** |
| **Level of Educational Obtainment** | | | | |
| Tertiary level educated | Ref | Ref | Ref | Ref |
| Trade certificate | **1.06 (1.02-1.11)**** | 1.12 (1.00-1.25) | 1.00 (0.99-1.01) | **1.38 (1.10-1.74)**** |
| High School Certificate | 1.02 (0.98-1.06) | 0.98 (0.88-1.10) | 1.01 (1.00-1.02) | 1.13 (0.90-1.42) |
| Did not finish high school | **1.06 (1.01-1.11)*** | 1.13 (1.00-1.29) | 0.99 (0.97-1.01) | **1.73 (1.35-2.20)***** |
| Still in school | 1.09 (0.96-1.23) | 1.18 (0.89-1.56) | 0.99 (0.95-1.03) | 1.24 (0.72-2.15) |
| **Self-assessed health** | | | | |
| Excellent | Ref | Ref | Ref | Ref |
| Very good | **1.09 (1.05-1.14)***** | **1.29 (1.13-1.47)***** | **0.98 (0.97-0.99)***** | 1.00 (0.79-1.26) |
| Good | **1.21 (1.16-1.27)***** | **1.72 (1.50-1.96)***** | **0.95 (0.94-0.96)***** | **1.52 (1.20-1.91)***** |
| Fair | **1.31 (1.24-1.39)***** | **2.00 (1.72-2.33)***** | **0.94 (0.92-0.96)***** | **1.52 (1.16-1.98)**** |
| Poor | **1.30 (1.16-1.45)***** | **1.82 (1.43-2.30)***** | **0.93 (0.88-0.98)**** | **1.80 (1.21-2.70)**** |
| **Number of people in dwelling**  (Multi person household) | 0.99 (0.98-1.00) | 1.05 (0.94-1.16) | **1.00 (0.99-1.00)*** | 0.98 (0.80-1.19) |
| **Working Status** | | | | |
| Employed | Ref | Ref | Ref | Ref |
| Unemployed, looking for work | **1.13 (1.07-1.19)***** | **1.28 (1.14-1.44)***** | **0.97 (0.95-0.99)**** | **1.23 (1.00-1.50)*** |
| Unemployed, not looking for work | **1.11 (1.07-1.15)***** | **1.26 (1.15-1.37)***** | **0.97 (0.96-0.98)***** | **1.27 (1.08-1.49)**** |
| **Gross Annual Household Income**  (Above Median) | **1.00 (1.00-1.00)*** | **1.16 (1.07-1.26)***** | **1.00 (1.00-1.00)**** | 1.05 (0.92-1.21) |
| ***Community Participation Variables*** | | | | |
| **Civic Engagement**  (Top three quartiles) | **1.04 (1.02-1.06)***** | 0.93 (0.86-1.00) | **0.99 (0.98-1.00)*** | 0.88 (0.77-1.02) |
| **Community Engagement**  (Top three quartiles) | **0.93 (0.92-0.95)***** | **1.34 (1.25-1.45)***** | **1.04 (1.03-1.04)***** | **1.58 (1.38-1.81)***** |
| **Altruism**  (Top three quartiles) | 0.99 (0.97-1.00) | 1.07 (0.99-1.16) | 1.00 (1.00-1.00) | 1.03 (0.90-1.19) |
| **Cultural Practices**  (Top three quartiles) | **0.97 (0.95-0.98)***** | **1.11 (1.02-1.20)*** | 1.00 (1.00-1.01) | 1.07 (0.93-1.22) |
| ***Neighbourhood Variables*** | | | | |
| **Neighbourhood Safety**  (Top three quartiles) | **0.96 (0.94-0.98)***** | **1.21 (1.12-1.30)***** | **1.01 (1.01-1.02)***** | **1.17 (1.02-1.35)*** |
| **Neighbourhood Social Cohesion**  (Top three quartiles) | 0.99 (0.97-1.00) | **1.07 (1.00-1.16)** | 1.00 (1.00-1.01) | **1.54 (1.34-1.76)***** |
| **Neighbourhood Atmosphere**  (Top three quartiles) | 0.99 (0.96-1.02) | 1.02 (0.94-1.10) | 1.00 (0.99-1.00) | 0.94 (0.83-1.08) |
| **Remoteness** | | | | |
| Major Cities | Ref | Ref | Ref | Ref |
| Inner Regional | **1.04 (1.00-1.08)*** | 1.08 (0.99-1.18) | **0.99 (0.97-1.00)*** | **1.29 (1.11-1.49)***** |
| Outer Regional- Very Remote | 1.04 (0.99-1.09) | **1.13 (1.02-1.26)*** | 1.00 (0.98-1.01) | 1.06 (0.87-1.30) |
| **SEIFA Index** | 1.00 (1.00-1.00) |  | 1.00 (1.00-1.00) |  |
| Low 1 |  | Ref |  | Ref |
| 2 |  | 0.98 (0.89-1.09) |  | 1.01 (0.85-1.20) |
| 3 |  | 0.99 (0.89-1.10) |  | 1.19 (0.99-1.43) |
| 4 |  | 1.04 (0.93-1.16) |  | 0.95 (0.77-1.17) |
| High 5 |  | 0.98 (0.86-1.10) |  | 1.07 (0.85-1.34) |
| ***Other Observations*** | | | | |
| **Time** | 1.00 (1.00-1.01) | **1.01 (1.00-1.02)** | 1.00 (1.00-1.00) | **1.01 (1.00-1.03)** |
| **Intercept** | **5.97 (4.73-7.54)***** | **0.07 (0.06-0.09)***** | **5.59 (5.20-6.00)***** | **0.02 (0.01-0.03)***** |
| **AIC** | 28785.43 | 9879.65 | 31458.66 | 5061.84 |
| **BIC** | 29023.90 | 10146.17 | 31683.12 | 5328.39 |

*** p<.001, ** p<.01, * p<.05

**Supp Table 3.** *Variance Inflation Factors (example from model for loneliness in older adults)*

| Variable | VIF |
| --- | --- |
| Social Isolation | 1.08 |
| Civic Engagement | 1.28 |
| Community Engagement | 1.25 |
| Altruism | 1.21 |
| Cultural Practices | 1.24 |
| Neighbourhood Safety | 1.16 |
| Neighbourhood Social Cohesion | 1.12 |
| Neighbourhood Atmosphere | 1.14 |
|  |  |
| Mean VIF | 1.19 |

**Supp Table 4.** *Correlation matrix of coefficients* *(example from model for loneliness in older adults)*

|  | Social Isolation | Civic Engagement | Community Engagement | Altruism | Cultural Practices | Neighbourhood Safety | Neighbourhood Social Cohesion | Neighbourhood Atmosphere |
| --- | --- | --- | --- | --- | --- | --- | --- | --- |
| Social Isolation | 1.0000 |  |  |  |  |  |  |  |
| Civic Engagement | -0.0125 | 1.0000 |  |  |  |  |  |  |
| Community Engagement | -0.2023 | -0.1467 | 1.0000 |  |  |  |  |  |
| Altruism | -0.0150 | -0.2422 | -0.1117 | 1.0000 |  |  |  |  |
| Cultural Practices | -0.0366 | -0.2347 | -0.1733 | -0.1433 | 1.0000 |  |  |  |
| Neighbourhood Safety | -0.0185 | 0.0389 | -0.0417 | 0.0027 | 0.0370 | 1.0000 |  |  |
| Neighbourhood Social Cohesion | -0.0872 | -0.0354 | -0.1082 | -0.0639 | -0.0609 | -0.1639 | 1.0000 |  |
| Neighbourhood Atmosphere | 0.0030 | 0.0005 | -0.0151 | 0.0013 | -0.0242 | -0.2999 | -0.0989 | 1.0000 |
