# Supplementary material for: Community-level determinants of loneliness and social isolation: a population-based cohort study across younger and older adults
Source: Front Public Health. 2025 May 15;13:1526166. doi: 10.3389/fpubh.2025.1526166 (PMC12119266; doi:10.3389/fpubh.2025.1526166)
Supplement: Supplementary file 5 [file Supplementary_file_5.docx]

**Supplementary File 5.**

**Supp. Table 1.** *Poisson regression of loneliness and social isolation displaying risk ratios for individual level covariates. Significant results are bolded.*

| **Variables** | **Loneliness** | | **Social Isolation** | |
| --- | --- | --- | --- | --- |
| **Population** | **Younger (18-30)** | **Older (60+)** | **Younger (18-30)** | **Older (60+)** |
|  | **RR (95% CI)** | **RR (95% CI)** | **RR (95% CI)** | **RR (95% CI)** |
| **Age** | | | | |
| 18-21 | Ref | - | Ref | - |
| 22-24 | 1.07 (0.98-1.17) | - | 0.98 (0.83-1.15) | - |
| 25-27 | 1.06 (0.95-1.17) | - | 1.09 (0.91-1.31) | - |
| 28-30 | 1.07 (0.96-1.19) | - | 1.00 (0.83-1.21) | - |
| 60-64 | - | Ref | - | Ref |
| 65-69 | - | 0.95 (0.87-1.04) | - | 0.96 (0.83-1.12) |
| 70-74 | - | **0.89 (0.81-0.99)*** | - | 1.02 (0.85-1.23) |
| 75-79 | - | 0.90 (0.81-1.02) | - | 1.01 (0.82-1.24) |
| 80-84 | - | 0.99 (0.87-1.13) | - | 1.09 (0.85-1.39) |
| 85-89 | - | 0.92 (0.77-1.10) | - | 0.78 (0.54-1.13) |
| 90+ | - | 0.92 (0.70-1.21) | - | 0.66 (0.33-1.32) |
| **Gender** | | | | |
| Male | Ref | Ref | Ref | Ref |
| Female | **1.21 (1.13-1.31)***** | **1.14 (1.05-1.23)**** | **0.69 (0.61-0.79)***** | **0.76 (0.66-0.88)***** |
| **Ethnicity** | | | | |
| Australian, non-Indigenous | Ref | Ref | Ref | Ref |
| Australian-Indigenous | 1.00 (0.86-1.15) | 1.27 (0.84-1.92) | 1.20 (0.96-1.49) | 1.55 (0.85-2.83) |
| Main English-speaking country born | **1.24 (1.03-1.49)*** | 1.03 (0.92-1.14) | 1.09 (0.75-1.60) | 1.02 (0.86-1.22) |
| Others | 1.05 (0.90-1.23) | **1.20 (1.08-1.33)***** | 1.08 (0.81-1.44) | 1.06 (0.89-1.27) |
| **Marital Status** | | | | |
| Legally married or de facto | Ref | Ref | Ref | Ref |
| Separated or divorced | 1.25 (0.94-1.66) | **1.33 (1.16-1.53)***** | **1.60 (1.03-2.49)*** | 1.16 (0. 91-1.47) |
| Widowed | - | **1.46 (1.27-1.68)***** | **-** | 1.17 (0.90-1.51) |
| Never married and not de facto | **1.36 (1.24-1.48)***** | **1.28 (1.06-1.54)*** | **1.24 (1.07-1.45)**** | 1.23 (0.91-1.68) |
| **Level of Educational Obtainment** | | | | |
| Tertiary level educated | Ref | Ref | Ref | Ref |
| Trade certificate | 1.12 (1.00-1.25) | 0.98 (0.87-1.11) | **1.38 (1.10-1.74)**** | 1.00 (0.82-1.22) |
| High School Certificate | 0.98 (0.88-1.10) | 1.04 (0.89-1.22) | 1.13 (0.90-1.42) | 0.97 (0.74-1.28) |
| Did not finish high school | 1.13 (1.00-1.29) | 1.00 (0.88-1.12) | **1.73 (1.35-2.20)***** | 0.88 (0.72-1.08) |
| Still in school | 1.18 (0.89-1.56) | - | 1.24 (0.72-2.15) | - |
| **Self-assessed health** | | | | |
| Excellent | Ref | Ref | Ref | Ref |
| Very good | **1.29 (1.13-1.47)***** | **1.36 (1.01-1.83)*** | 1.00 (0.79-1.26) | 1.27 (0.83-1.94) |
| Good | **1.72 (1.50-1.96)***** | **2.05 (1.54-2.74)***** | **1.52 (1.20-1.91)***** | 1.38 (0.91-2.09) |
| Fair | **2.00 (1.72-2.33)***** | **2.66 (2.00-3.55)***** | **1.52 (1.16-1.98)**** | **1.61 (1.06-2.46)*** |
| Poor | **1.82 (1.43-2.30)***** | **3.12 (2.31-4.21)***** | **1.80 (1.21-2.70)**** | **1.95 (1.24-3.07)**** |
| **Number of people in dwelling** | | | | |
| Multi-person household | Ref | Ref | Ref | Ref |
| Lone person household | 1.05 (0.94-1.16) | **1.15 (1.01-1.30)*** | 0.98 (0.80-1.19) | 1.05 (0.84-1.33) |
| **Working Status** | | | | |
| Employed | Ref | Ref | Ref | Ref |
| Unemployed-looking for work | **1.28 (1.14-1.44)***** | 1.10 (0.76-1.58) | **1.23 (1.00-1.50)*** | 0.95 (0.49-1.85) |
| Unemployed-not looking for work | **1.26 (1.15-1.37)***** | **1.11 (1.01-1.23)*** | **1.27 (1.08-1.49)**** | 1.02 (0.86-1.21) |
| **Gross Annual Household Income** | | | | |
| Above Median | Ref | Ref | Ref | Ref |
| Below Median | **1.16 (1.07-1.26)***** | 1.04 (0.94-1.15) | 1.05 (0.92-1.21) | 0.95 (0.80-1.12) |
| *** *p*<.001, ** *p*<.01, * *p*<.05 | | | | |

Note: All models adjusted for the following community participation and neighbourhood variables: civic engagement, community engagement, altruism, and cultural practices, neighbourhood safety, neighbourhood social cohesion, neighbourhood atmosphere, remoteness, and Socioeconomic Index for Area (SEIFA) quintiles.
